# Tailoring of Physical Properties in Macroporous Poly(isocyanopeptide) Cryogels

**DOI:** 10.1021/acs.biomac.4c00086

**Published:** 2024-05-14

**Authors:** Lotte Gerrits, Bram Bakker, Lynn D. Hendriks, Sjoerd Engels, Roel Hammink, Paul H. J. Kouwer

**Affiliations:** † Institute for Molecules and Materials, Radboud University, Heyendaalseweg 135, 6525 AJ Nijmegen, The Netherlands; ‡ Institute for Chemical Immunology, 6525 GA Nijmegen ,Netherlands; § Department of Medical BioSciences,Radboudumc, Geert Grooteplein 26, 6525 GA Nijmegen, The Netherlands; ∥ Division of Immunotherapy, Oncode Institute, Radboud University Medical Center, 6525 GA Nijmegen ,Netherlands

## Abstract

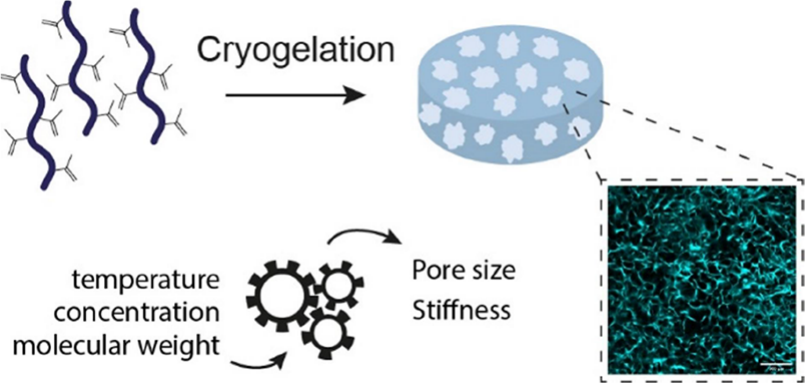

Over the years, synthetic hydrogels have proven remarkably useful as cell culture matrixes to elucidate the role of the extracellular matrix (ECM) on cell behavior. Yet, their lack of interconnected macropores undermines the widespread use of hydrogels in biomedical applications. To overcome this limitation, cryogels, a class of macroporous hydrogels, are rapidly emerging. Here, we introduce a new, highly elastic, and tunable synthetic cryogel, based on poly(isocyanopeptides) (PIC). Introduction of methacrylate groups on PIC facilitated cryopolymerization through free-radical polymerization and afforded cryogels with an interconnected macroporous structure. We investigated which cryogelation parameters can be used to tune the architectural and mechanical properties of the PIC cryogels by systematically altering cryopolymerization temperature, polymer concentration, and polymer molecular weight. We show that for decreasing cryopolymerization temperatures, there is a correlation between cryogel pore size and stiffness. More importantly, we demonstrate that by simply varying the polymer concentration, we can selectively tune the compressive strength of PIC cryogels without affecting their architecture. This unique feature is highly useful for biomedical applications, as it facilitates decoupling of stiffness from other variables such as pore size. As such, PIC cryogels provide an interesting new biomaterial for scientists to unravel the role of the ECM in cellular functions.

## Introduction

Hydrogels are cross-linked polymer networks that have the ability to absorb large amounts of water without losing their structural integrity. Typically, their tunable biochemical and biophysical properties as well as their resemblance with the natural extracellular matrix (ECM) makes hydrogels attractive to use as cell culture matrix for tissue engineering and regenerative medicine purposes.^1−5^ It is well established that the mechanical characteristics of the matrix play a crucial role in dictating cellular functions, such as cell spreading, differentiation, and proliferation.^6−10^ The stiffness of hydrogels is readily adjusted by changing the degree of cross-linking or the concentration of gel precursors, or by the addition of nanomaterials or preparation of interpenetrating polymer networks (IPNs).^5,11−14^ Besides the stiffness, however, these strategies simultaneously affect the gel architecture is another essential factor that dictates cell behavior.^15−17^ Consequently, (bio)materials scientists direct much of their efforts to decouple stiffness from gel architecture when investigating how the ECM regulates cell behavior.

Poly(isocyanopeptide) (PIC)-based hydrogels form a class of fully synthetic materials for which the stiffness and architecture can be readily decoupled.^18,19^ PIC polymers are built helical polymers that form thermoreversible gels in aqueous solutions; at low temperatures, the polymer dissolves, and upon heating, they bundle together to form a gel with a fibrous architecture that is similar to biogels such as collagen and fibrin.^20−22^

Additionally, PIC hydrogels exhibit mechanical properties that closely mimic the native cell environment.^20^ A recent study demonstrated that in terms of mechanics and architecture, there is tremendous overlap between PIC hydrogels and collagen gels.^23^ Furthermore, the PIC gels are readily functionalized with biochemical cues through bio-orthogonal click chemistry between the azide groups on the polymer and DBCO-modified biomolecules.^24^ The combination of tailorable mechanics and biochemical properties makes PIC hydrogels attractive for a range of biomedical applications.^25−33^

Synthetic matrices such as PIC hydrogels rely on the physical remodeling mechanism to facilitate cellular processes such as proliferation and migration.^23,34−38^ While in physically cross-linked matrices, such remodeling process may be feasible for adherent cells, nonadherent cells, such as hematopoietic cells including T-cells, cannot remodel their matrix so easily. They depend on a macroporous architecture that intrinsically supports cell proliferation and migration.^39^ Additionally, macro-sized pores provide a larger surface area per unit volume, which facilitates diffusion of metabolites and nutrients.^40^ In this article, we present strategies to expand the characteristic micrometer-sized porous architecture of the PIC gels^19^ to tens of micrometers without compromising their unique mechanical properties.

A variety of methods can be employed to prepare macroporous hydrogels, including porogen templating,^41,42^ gas foaming,^43,44^ freeze-drying,^45^ 3D printing,^46,47^ and cryogelation.^48−51^ In the last method, a hydrogel precursor solution is cooled to subzero temperatures, causing a large part of the solvent to crystallize, which forces gel forming components such as monomers and polymers to concentrate in the nonfrozen microphase, where chemical cross-linking takes place.^52^ The so-called cryo-concentration of these constituents accelerates the formation of a macroporous gel network.^53^ Subsequent thawing of the ice crystals results in the formation of a highly interconnected macroporous hydrogel network. Since the ice crystals function as porogens, there is no need to remove potentially harmful micrometer-sized templates.^54−56^ Hydrogels formed via cryogelation are termed cryogels^57^ and examples based on alginate, gelatin, silk or composites thereof have been successfully used for tissue engineering applications.^50,58,59^

Here, we develop a synthetic cryogel from the PIC scaffolds. We investigate how cryogelation parameters^55,56^ such as polymerization temperature, freezing rate, and polymer concentration, can be utilized to tune the architectural and mechanical characteristics of the resulting cryogels. Prepared cryogels typically have a highly interconnected macroporous structure and exhibit swelling and shape memory behaviors that are characteristic for cryogels. We find that the PIC cryogels polymerized at lower temperatures showed smaller pores and a higher compressive strength. Compositional changes, however, primarily affect the mechanical properties and not the pore size, which are unique for PIC-based cryogels. In short, PIC cryogels are a new and highly tailorable class of cryogels that can be used in biomedical applications in which the influence of pore size and matrix stiffness is of high importance.

## Materials and Methods

### Synthesis Methacrylate-Functionalized Isocyanopeptide Monomer

The methacrylate-functionalized isocyanopeptide monomer was synthesized via a protocol similar to the protocol described in the literature for synthesis of the methoxy-functionalized isocyanopeptide monomer.^60^ Instead of starting with tetraethylene glycol, the synthesis route was started with tetraethylene glycol monobenzyl ether, which was deprotected and functionalized with a methacrylate group. The divergent steps in the synthesis route are described below. An overview of the full synthesis route is depicted in Scheme S1 (Supporting Information).

### Synthesis of Tetraethylene Glycol Monobenzyl Ether (**1a**)

Tetraethylene glycol (TEG) (17.26 mL, 100 mmol) was added dropwise to a cooled (0 °C) solution of NaH (1.03 g 60% in mineral oil, 25.8 mmol) in tetrahydrofuran (75 mL) under Schlenk conditions and stirred for 45 min. A solution of BnBr (4.490 g, 25.8 mmol) in THF (125 mL) was added, and the resulting mixture was warmed to r.t. The reaction mixture was stirred for 72 h at r.t. and subsequently concentrated *in vacuo.* The crude product was dissolved in EtOAc (100 mL) and washed with water (3 × 50 mL). The combined aqueous layers were washed with EtOAc (3 × 50 mL). Combined organic layers were dried with NaSO_4_ and concentrated *in vacuo*. The product was isolated via column chromatography (SiO_2_, EtOAc) to afford **1a** as a yellow oil (5.16 g, 18.2 mmol, 70.3%). **TLC** (EtOAc): *R*_f_ = 0.46, ^**1**^**H NMR** (400 MHz, CDCl_3_) δ 7.32–7.14 (m, 5H, Ar-*H*), 4.48 (s, 2H, Ar–C*H*2), 3.69–3.47 (m, 16H, OC*H*_2_), 2.80 (s, 1H, -O*H*). ^**13**^**C NMR** (101 MHz, CDCl_3_) δ 138.06 (Ar-*C*CH_2_), 128.17 (Ar-*C*H), 127.56 (Ar-*C*H), 127.39 (Ar-*C*H), 73.02 (Ar-*C*H_2_), 72.37 (O*C*H_2_), 70.44 (O*C*H_2_), 70.41 (O*C*H_2_), 70.38 (O*C*H_2_), 70.14 (O*C*H_2_), 69.25 (O*C*H_2_), 61.47 (O*C*H_2_).

### Synthesis of 1-Phenyl-2,5,8,11-tetraoxatridecan-13-yl Formyl-*D*-Alanyl-*L*-Alaninate (**2**)

The following steps toward synthesis of the methacrylate monomer are previously described in the literature.^60^ In short, a Boc-protected l-alanine ester was formed through reaction of **1a** with Boc-l-Ala using 4-dimethylaminopyridine (DMAP) and N,*N*′-dicyclohexylcarbodi-imide (DCC) as coupling reagents (affording **1b**, Scheme S1). After Boc-deprotection using HCl (4 M in Dioxane), a similar condensation reaction with Boc-d-Alanine was performed using DMAP, DCC and *N*-hydroxybenzotriazole (HOBt) (resulting in **1c**, Scheme S1). Subsequent deprotection with HCl (4 M in dioxane) afforded the free amine, which was formylated with ethyl formate to afford **2** as a yellow oil (626 mg, 1.38 mmol, 26% over 5 steps). ^**1**^**H NMR** (400 MHz, CDCl_3_) δ 8.18 (s, 1H, *H*C = ONH), 7.35–7.24 (m, 5H, Ar-*H*), 6.85 (d, *J* = 7.8 Hz, 1H, N*H*), 6.66 (d, *J* = 7.9 Hz, 1H, N*H*), 4.64–4.51 (m, 4H, Ar–C*H*_*2*_, 2 × C*H*CH_3_), 4.36–4.18 (m, 2H, OC*H*_2_), 3.72–3.61 (m, 14H, OC*H*_2_), 1.40 (ddd, *J* = 16.3, 7.1, 0.9 Hz, 6H, 2 × C*H*_*3*_). ^**13**^**C NMR** (101 MHz, CDCl_3_) δ 172.43 (*C*=OCHCH_3_), 171.32 (*C*=OCHCH_3_), 161.33 (H*C*=ONH), 138.12 (Ar-*C*CH_2_), 128.37 (Ar-*C*H), 127.75 (Ar-*C*H), 127.65 (Ar-*C*H), 73.23 (Ar-*C*H_2_), 70.63 (O*C*H_2_), 70.55 (O*C*H_2_), 70.53 (O*C*H_2_), 70.51 (O*C*H_2_), 70.48 (O*C*H_2_), 69.39 (O*C*H_2_), 68.94 (O*C*H_2_), 64.41 (O*C*H_2_), 48.35 (*C*HCH_3_), 47.12 (*C*HCH_3_), 17.90 (*C*H_3_), 17.56 (*C*H_3_).

### Synthesis of 2-(2-(2-(2-Hydroxyethoxy)ethoxy)ethoxy)ethyl Formyl-*D*-Alanyl-*L*-Alaninate (**3**)

Palladium catalyst (60.6 mg, 10% Pd/C) was added to a solution of **2** (600 mg, 1.32 mmol) in ethanol (25.0 mL). The resulting mixture was stirred under a H_2_ flow at r.t. for 18 h. The mixture was filtered over Celite, washed with ethanol, and concentrated *in vacuo* to yield **3** as a yellow oil (450 mg, 1.23 mmol). **TLC** (MeOH: DCM, 1:20, v/v): *R*_f_ = 0.23. ^**1**^**H NMR** (400 MHz, CDCl_3_) δ 8.20 (s, 1H, *H*C=ONH), 7.14 (d, *J* = 7.8 Hz, 1H, N*H*), 6.79 (s, 1H, N*H*), 4.69–4.54 (m, 2H, 2 × C*H*CH_3_), 4.29 (ddd, *J* = 5.9, 3.4, 1.3 Hz, 2H, OC*H*_2_), 3.77–3.59 (m, 14H, OC*H*_2_), 1.42 (dd, *J* = 11.5, 7.1 Hz, 6H, 2 × C*H*_*3*_). ^**13**^**C NMR** (101 MHz, CDCl_3_): δ 172.35 (*C*=OCHCH_3_), 171.49 (*C*=OCHCH_3_), 161.25 (H*C*=ONH), 72.49 (O*C*H_2_), 70.45 (O*C*H_2_), 70.44 (O*C*H_2_), 70.42 (O*C*H_2_), 70.10 (O*C*H_2_), 69.00 (O*C*H_2_), 64.25 (O*C*H_2_), 61.57 (O*C*H_2_), 48.32 (*C*HCH_3_), 47.16 (*C*HCH_3_), 18.04 (*C*H_3_), 17.92 (*C*H_3_).

### Synthesis of (3*R*,6*S*)-3,6-Dimethyl-1,4,7-trioxo-8,11,14,17-tetraoxa-2,5-diazanonadecan-19-yl Methacrylate (**4**)

To a solution of **3** (260 mg, 0.71 mmol) in dry dichloromethane (DCM, 18.0 mL), sodium methacrylate (386 mg, 3.60 mmol), di-isopropylethylamine (DIPEA) (184 mg, 1.40 mmol), and DMAP (43.6 mg, 0.360 mmol) were added, and the resulting mixture was cooled to 0 °C. A solution of *N*-(3-(dimethylamino)propyl)-*N′*-ethylcarbodiimide hydrochloride (EDC-HCl, 684 mg, 3.60 mmol) in DCM (12.0 mL) was added dropwise to the mixture and allowed to warm to rt. After stirring for 18 h, the reaction mixture was washed with an acidic brine solution (1 M HCl, 3 × 50 mL) and sat. NaHCO_3_ (3 × 50 mL). The combined organic layers were dried with Na_2_SO_4_, filtered, and concentrated *in vacuo* to afford **4** as a yellow oil (174 mg, 0.40 mmol). **TLC:** (MeOH: DCM, 1:10 v/v): *R*_f_ = 0.22. ^**1**^**H NMR** (400 Hz, CDCl_3_): δ 8.18 (s, 1H, *H*C=ONH), 6.93 (d, *J* = 7.7 Hz, 1H, N*H*), 6.70 (d, *J* = 7.8 Hz, 1H, N*H*), 6.13 (s, 1H, C = C*H*_*2*_), 5.61–5.54 (m, 1H, C = C*H*_*2*_), 4.68–4.51 (m, 2H, 2 × C*H*CH_3_), 4.36–4.20 (m, 4H, OC*H*_2_), 3.74 (ddd, *J* = 5.8, 3.7, 0.9 Hz, 2H, OC*H*_2_), 3.66 (dddt, *J* = 10.2, 6.4, 2.4, 1.4 Hz, 10H, OC*H*_2_), 1.98–1.92 (m, 3H, CCH_2_C*H*_*3*_), 1.41 (ddd, *J* = 11.6, 7.1, 0.9 Hz, 6H, 2 × CH_3_). ^**13**^**C NMR** (101 MHz, CDCl_3_): δ 172.46 (*C*=OCHCH_3_), 171.36 (*C*=OCHCH_3_), 167.33 (*C*=OC=CH_2_), 161.20 (H*C*=ONH), 136.09 (*C*=CH_2_), 125.77 (C=*C*H_2_), 70.61 (O*C*H_2_), 70.52 (O*C*H_2_), 70.49 (O*C*H_2_), 70.47 (O*C*H_2_), 69.08 (O*C*H_2_), 68.92 (O*C*H_2_), 64.37 (O*C*H_2_), 63.75 (O*C*H_2_), 48.29 (*C*HCH_3_), 47.13 (*C*HCH_3_), 18.27 (C=CH_2_*C*H_3_), 17.89 (*C*H_3_), 17.75 (*C*H_3_).

### Synthesis of (14*S*,17*R*)-17-Isocyano-14-methyl-13,16-dioxo-3,6,9,12-tetraoxa-15-azaoctadecyl Methacrylate (**5**)

A solution of the Burgess reagent (121 mg, 0.510 mmol) in dry DCM (15.0 mL) was added dropwise to a solution of **4** (169 mg, 0.390 mmol) in dry DCM (20.0 mL) under nitrogen flow. The resulting reaction mixture was stirred at rt for 6h and concentrated *in vacuo*. The crude oil was purified by using column chromatography (SiO_2_, 1 → 20% MeOH in DCM, v/v) to afford **4** as a yellow oil (95 mg, 0.23 mmol). **TLC** (MeOH: DCM, 1:10, v/v): *R*_f_ = 0.46. ^**1**^**H NMR** (400 Hz, CDCl_3_): δ 6.98 (d, *J* = 7.5 Hz, 1H, N*H*), 6.13 (dt, *J* = 2.0, 1.0 Hz, 1H, C=C*H*_*2*_), 5.58 (p, *J* = 1.6 Hz, 1H, C=C*H*_*2*_), 4.59 (p, *J* = 7.2 Hz, 1H, C*H*CH_3_), 4.38–4.22 (m, 5H, C*H*CH_3_, 2 × OC*H*_2_), 3.78–3.70 (m, 4H, OC*H*_2_), 3.69–3.61 (m, 8H, OC*H*_2_), 1.95 (dd, *J* = 1.6, 1.0 Hz, 3H, CCH_2_C*H*_*3*_), 1.65 (d, *J* = 3.1 Hz, 3H, CH_3_), 1.48 (d, *J* = 7.2 Hz, 3H, CH_3_). ^**13**^**C NMR** (101 MHz, CDCl_3_): δ 171.98 (*C*=OCHCH_3_), 167.35 (*C*=OC=CH_2_), 165.71 (*C*=OCHCH_3_), 136.16 (*C*=CH_2_), 125.73 (C=*C*H_2_), 70.63 (O*C*H_2_), 69.14 (O*C*H_2_), 68.82 (O*C*H_2_), 64.69 (O*C*H_2_), 63.82 (O*C*H_2_), 53.40 (*C*HCH_3_), 48.56 (*C*HCH_3_), 19.66 (C=CH_2_*C*H_3_), 18.30 (*C*H_3_), 18.03 (*C*H_3_). **MS** (ESI) *m*/*z*: found 437.10 (M + Na^+^), calcd 437.20.

### Synthesis of Methacrylate-Functionalized PIC

Methacrylate-functionalized PICs of varying lengths were synthesized following a similar protocol as described in the literature,^60^ with the only difference that a methacrylate monomer is added. In short, PICs comprising azide, methacrylate, and methoxy groups were synthesized. To this end, azide- and methacrylate-terminated isocyanide monomers were prepared as described in the literature^60^ (for azide monomers) or as described previously (for methacrylate monomers), while the commercially available methoxy-terminated monomers (Chiralix) were purified via column chromatography (SiO_2_, MeOH:DCM, 5:95, v/v) before use. Stock solutions of azide-terminated (1.29 mL, 10.3 mM), methacrylate-terminated (0.693 mL, 40.1 mM), and methoxy-terminated monomer (7.35 mL, 189 mM) in dry toluene, MBraun SPS 800 Solvent system) were combined. Dry toluene was added to reach a final concentration of 55 mg of monomers per mL. To obtain polymers with various lengths, three polymerization reactions were carried out with catalyst-to-monomer ratios of 1:1000 (**P1**), 1:3000 (**P2**), and 1:10,000 (**P3**), respectively. A solution of Ni(ClO_4_)_2_·6H_2_O (1:9, EtOH: toluene, v/v) was added to the reaction mixtures (584 μL, 2.45 mM for **P1**, 167 μL, 2.84 mM for **P2,** and 130 μL, 1.10 mM for **P3**). The resulting mixtures were reacted for 18 h at rt. Isocyanide consumption was confirmed by the disappearance of the characteristic FT-IR peak at 2140 cm^–1^. The polymers were precipitated three times in cold (0 °C) diisopropylether and dried overnight to yield **P1** as an off-white solid (1.35 g, 87%), **P2** as an off-white solid (1.16 g, 78%) and **P3** as an off-white solid (404 mg, 77%). Average polymer lengths were determined using atomic force microscopy (AFM, Nanoscope IV Bruker, NSG-10 tapping mode tips, NT-MDT) and were found to be 210 ± 138 nm (**P1**), 346 ± 231 nm (**P2**), and 562 ± 390 nm (**P3**) (Mean ± SD, Figure S1a–f). The characteristic helical backbone of the **P1**, **P2,** and **P3** was confirmed by circular dichroism spectroscopy of polymer solutions in Milli-Q (0.2 mg/mL) (Figure S1g).^61^

### Preparation of PIC Cryogels

Unless specified otherwise, cryogels were made using the following protocol. Stock solutions of PIC (**P1**, 833 μL, 11.0 mg/mL), 2-hydroxylethyl methacrylate (HEMA, Sigma-Aldrich) (97 μL, 7.33 mg/mL), and *N*,*N*,*N′*,*N′*-tetramethyl ethylenediamine (TEMED, Sigma-Aldrich) (20.0 μL, 58.0 mg/mL) in Milli-Q were combined and cooled on ice (0 °C) for 1 h prior to use. A cooled (0 °C) solution of ammonium persulfate (APS, Sigma-Aldrich) (50 μL, 100 mg/mL in Milli-Q) was added, and the reaction mixture was mixed by gently pipetting up and down and transferred to a custom-made Teflon mold (200 μL per well) that was cooled on ice (0 °C) for 1 h before use. After filling of each well, the mold was placed in a cryostat (−20 °C, SLEE Medical, MEV) for 18 h. The mold was then removed from the cryostat, and Milli-Q was added to thaw the cryogels. The cryogels were washed with Milli-Q and stored in the refrigerator (5 °C) until further use.

For synthesis of cryogels with varying polymer concentrations, stock solutions of **P1** (4.50, 8.00, and 11.0 mg/mL) in Milli-Q were used, and the amount of HEMA added was adjusted accordingly by using HEMA stock solutions of 3.00, 5.33, and 7.33 mg/mL, respectively. TEMED and APS were added by following the general protocol. For synthesis of cryogels consisting of polymers with different lengths, stock solutions of **P1**, **P2,** or **P3** (all 4.5 mg/mL) in Milli-Q were made, and the amount of HEMA added was adjusted accordingly (3.00 mg/mL). TEMED and APS were added following the general protocol.

### Cryogel Pore Size Analysis

Cryogels were labeled with AzDye 647 through addition of dibenzocyclooctyne (DBCO)-functionalized AzDye 647 (Click Chemistry Tools) as follows: cryogels were dehydrated using a medical gauze (kliniray gauze compress X-ray) and submerged in a solution of AzDye 647 DBCO (0.25 equiv rt azides in cryogel, 0.250 mL, 0.051 mM) in Milli-Q. After incubation for 1 h at rt, gels were washed three times with 0.05% PBS Tween, three times with PBS and submerged in PBS. Confocal microscopy was performed on hydrated cryogels using a Leica SP8x AOBS-WLL microscope. Per cryogel, three z-stacks (30 slices) were recorded. Pore sizes were determined from z-stack analysis in Fiji.^62^ Z-stacks were segmented using the Trainable Weka Segmentation plugin,^63^ followed by determination of the pore sizes using the BoneJ plugin.^64^

### Determination of Mechanical Properties of the Cryogels

Hydrated cryogels were subjected to uniaxial compression tests on a Discovery HR-2 (TA Instruments), using a 20 mm steel Peltier plate at 20 °C. Cryogels were compressed at a constant linear rate of 10 μm/s. The axial force and displacement data were used to obtain the stress–strain curves. The compressive stress (σ) was calculated from the recorded axial force (*F*) per cross-sectional area (*A*) of the undeformed sample. The strain (ε) was determined by calculating the ratio between the deformed (d*l*) and initial (*l*) lengths. Young’s modulus (also known as compression modulus, *E*) was calculated from the slope of the stress–strain curves at 80% strain via eq 1:
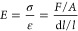
1

### Determination of Cryogel Swelling Ratio

To analyze the swelling behavior of the prepared cryogels, lyophilized cryogels (*m*_dry_) were weighed before immersion in Milli-Q. After 5 min incubation to allow the cryogels to swell, excess water was removed, and the cryogel weight (*m*_wet_) was determined. Then, the swelling ratio was calculated via eq 2.
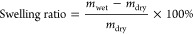
2

## Results and Discussion

### Synthesis and Characterization of Methacrylate PIC Scaffolds

**Figure 1:**
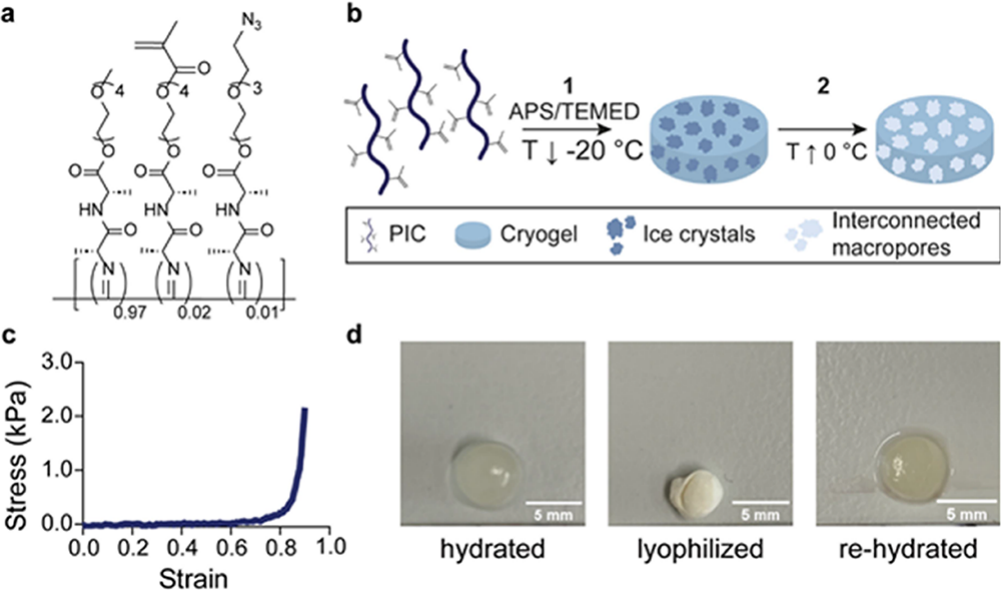
Preparation of PIC cryogels with shape memory. (a) Chemical structure of the PIC scaffold. (b) Overview of the preparation of PIC cryogels. Cryogels were synthesized from methacrylate-functionalized PIC scaffolds. (1) APS and TEMED were added as initiator systems to an aqueous solution of PIC to induce cross-linking at −20 °C. (2) Thawing of the ice crystals gives rise to an interconnected macroporous cryogel architecture. (c) Stress–strain curve of a PIC cryogel. This curve was obtained through uniaxial compression of the PIC cryogel until 90% strain. (d) Lyophilized cryogels regain their original size and shape after rehydration.

The cross-linking mechanism in the PIC cryogels follows the commonly used free-radical polymerization approach, which requires methacrylate groups to be introduced in the polymer. As such, we designed a new methacrylate-containing isocyanide monomer and randomly copolymerized^60^ it with the default PIC monomer. In addition, we introduced the azide (N_3_)-appended monomer that is used in our group for postmodification with cell-adhesive peptides or, in this case, with fluorescent dyes (Figure 1a). The azide and methoxy-functionalized monomers were obtained via reported procedures.^60^ A new synthesis route was developed to obtain the methacrylate-functionalized monomer (Scheme S1). In short, one hydroxyl of tetraethylene glycol was protected with a benzyl group, which was followed by two alanine couplings and subsequent formylation of the amine. The hydroxyl was then deprotected via hydrogenation, and methacrylic acid was conjugated through a condensation reaction. Dehydration of the formyl group afforded the isocyanide-methacrylate monomer.

Copolymerization of the monomer mixture (in different ratios) using a Ni^2+^ catalyst yielded random copolymers. PIC scaffolds with methacrylate fractions greater than 0.02 were insoluble in aqueous solutions, which is required for cryogelation. Based on this result, we continued with PICs with a methacrylate and azide fraction of 0.02 and 0.01, respectively. Polymers with different lengths were synthesized by varying the catalyst to monomer ratio: 1:1000 for **P1**, 1:3000 for **P2** and 1:10,000 for **P3**, which yielded polymers of average lengths of 209, 346, and 562 nm, respectively, as determined using atomic force microscopy (Figure S1a–f). The characteristic thermoresponsive behavior of PICs^20^ was demonstrated with rheology, and we found gelation temperatures of 47, 41, and 40 °C for **P1**, **P2,** and **P3**, respectively (Figure S2), which is in agreement with the previously reported notion that PICs with shorter lengths have a higher gelation temperature.^22^

### Preparation and Analysis of PIC Cryogels

After the successful synthesis of methacrylate polymers, we set out to develop PIC-based cryogels via a cryopolymerization process using free-radical polymerization (Figure 1b). In a typical experiment, an aqueous reaction mixture containing methacrylate-functionalized polymers and cross-linking agents (ammonium persulfate (APS) and tetramethylethylenediamine (TEMED)) was cooled to the polymerization temperature (e.g., −20 °C), and the cross-linking reactions was allowed to take place overnight. Then, the ice crystals were thawed and the cryogel was thoroughly washed with water to remove unreacted residual ingredients. The mechanical properties of the cryogels were studied in compression mode, where a freshly fully hydrated gel was subjected to uniaxial compression up to 90% (without breaking), which resulted in stress–strain curves, like that in Figure 1c, that demonstrate the elastic and ductile nature of the cryogel. From the stress–strain curves, we determine Young’s modulus *E*′ at a fixed strain (ε = 80%). PIC cryogels can undergo multiple rounds of compression without losing their mechanical properties (Figure S3). Because of its interconnected and macroporous structure, the PIC cryogel is sponge-like and exhibits shape memory behavior. After lyophilization, which shrinks the gel, the cryogel regains its original shape when rehydrated (Figure 1d). The architecture of the cryogels were studied by determining the cryogel swelling ratio, which is a measure for porosity,^65^ and by confocal fluorescence microscopy after labeling the cryogels with a fluorescent dye. From the latter experiment, average pore sizes and pore size distributions were calculated.

### Initiator and Comonomer Concentrations

Before we set out to investigate which parameters in the cryogelation process could be employed to tune the properties of PIC cryogels, we determined the optimal concentration initiators required for cryogel formation. At APS and TEMED concentrations below 0.02 and 0.01 mM, respectively, the formed PIC cryogels were weak and disintegrated upon handling, indicating that the degree of polymer cross-linking was insufficient for cryogel formation (Figure S4a–c). Beyond these concentrations, however, the formed cryogels were mechanically stable. Based on these findings, we continued cryopolymerization with 0.02 mM APS and 0.01 mM TEMED.

**Figure 2:**
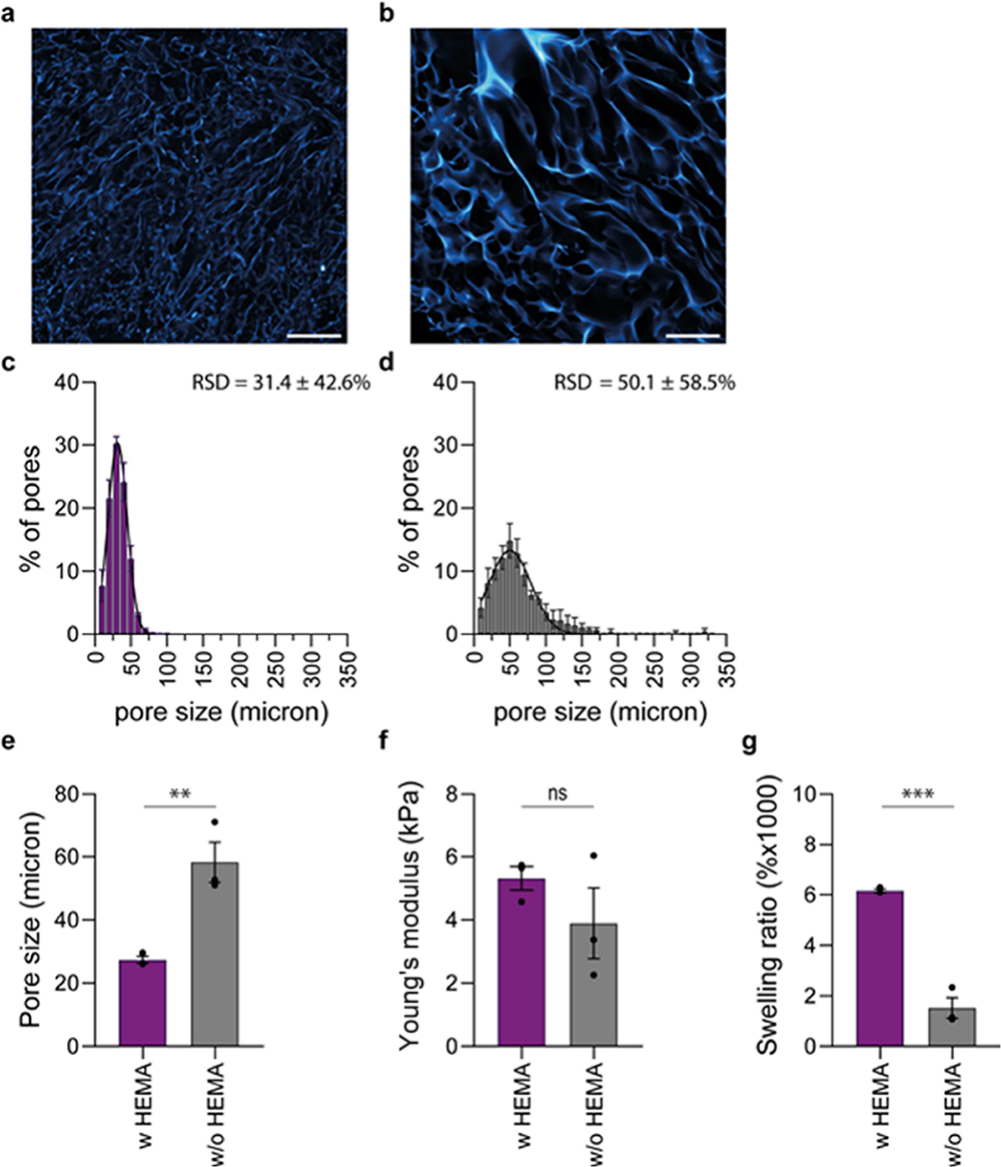
Addition of comonomer (HEMA) influences architectural and mechanical properties of PIC cryogels. (a and b) Confocal images showing the interconnected macroporous structure of PIC cryogels prepared with (a) and without (b) comonomer (HEMA). Scale bars are 200 μm. (c and d) Pore size distribution of PIC cryogels prepared with (c) and without (d) comonomer (HEMA). (e) Average pore sizes of PIC cryogels. Pore sizes were determined from z-stacks of CLSM images. Per condition, three cryogels were imaged, and for each cryogel, three z-stacks were analyzed to obtain an average pore size per cryogel. (f) Young's moduli of PIC cryogels prepared with (a) and without (b) comonomer (HEMA). Young's moduli were calculated from the stress strain curves that were obtained by compressing the cryogels. A derivative of the slope at 0.8 strain was taken to calculate Young’s moduli depicted in (f). (g) Swelling ratio of PIC cryogels prepared with (a) and without (b) comonomer (HEMA). All data are represented as mean ± SEM. Each dot represents a cryogel. (e–h) Statistical significance was tested with an unpaired *t* test. Stars indicate significance levels ** *P* ≤ 0.01, *** *P* ≤ 0.001, and ns is nonsignificant.

To further establish the optimal composition of PIC cryogels, we investigated the influence of the addition of an acrylate chain extender on the architectural and mechanical cryogel properties. It is well-known that the ratio between chain extender and polymer within a cryogel affects properties, such as pore size and swelling ratio.^66,67^ Here, we added 2-hydroxylethyl methacrylate (HEMA) as a chain extender to the PIC cryogel reaction mixture. Cryogels that were prepared with HEMA had a smaller average pore size (27 μm) than cryogels that were prepared without HEMA (58 μm; Figure 2a,b,e). The distribution of the pore sizes was narrower for cryogels that contained HEMA than for cryogels without HEMA (Figure 2c,d), which suggests that the addition of HEMA results in cryogels with a more homogeneous architecture.^68^ A z-stack of confocal images displaying an entire cryogel confirmed the uniformity of the pores throughout the gel (Video S1). Uniaxial compression tests (Figure S4) showed that cryogels prepared with HEMA display a slightly higher Young’s modulus (5.3 kPa) than those prepared without HEMA (3.9 kPa) (Figure 2f). While the difference is not significant, the observed trend is in line with previously described research where the addition of a chain extender resulted in cryogels with a higher compressive strength.^69,70^

In the swelling ratio experiments, we observe a trend that is opposite of what is previously reported. While for most cryogels, the addition of an acrylate comonomer decreases the swelling ratio,^69,71^ we observe that cryogels containing HEMA exhibit a much higher swelling ratio (6149%) than cryogels without HEMA (1517%) (Figure 2g). The degree of cross-linking within a cryogel network can affect the density of the polymer walls in the cryogel, which in turn can affect the ability of a cryogel to absorb water.^56^ Usually, higher swelling ratios are associated with cryogels with lower degree of cross-linking and lower density of polymer walls.^48,55^ However, the observed increase in swelling ratio for PIC cryogels with HEMA could not be attributed to the cryogels’ polymer wall thickness (Figure S5). Instead, we attribute the increased swelling ratio to the introduction of HEMA groups in the cryogel network by the addition of HEMA, which is known to influence network swelling.^72,73^ As the addition of HEMA gave rise to cryogels with a homogeneous interconnected macroporous structure and excellent swelling behavior, we continued including HEMA in the preparation of subsequent cryogels.

### Cryogelation Temperature Influences Cryogel Architecture and Stiffness

**Figure 3:**
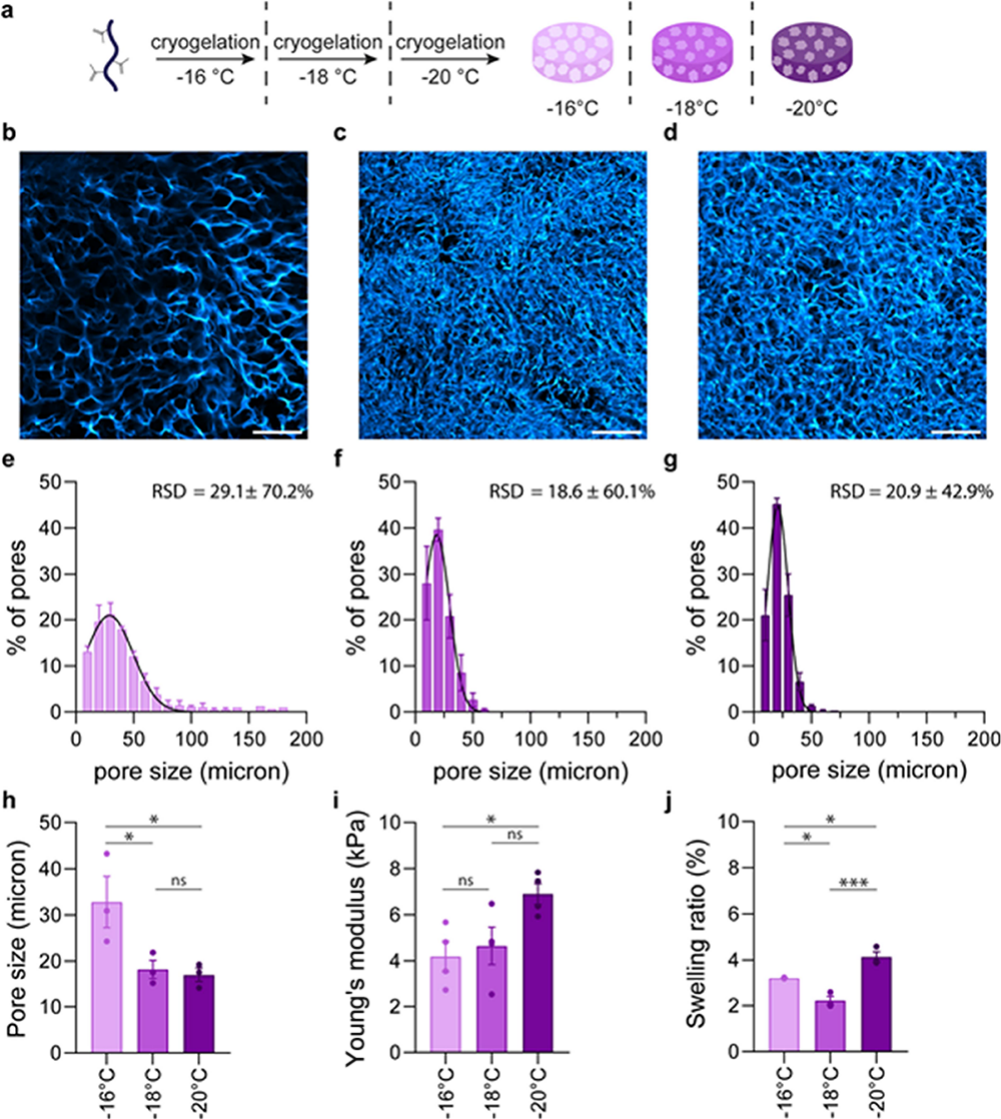
Cryogelation temperature influences architectural and mechanical properties of PIC cryogels. (a) Schematic overview of cryogel synthesis at different temperatures. (b,c) Confocal images showing the interconnected macroporous structure of PIC cryogels prepared at −16 °C (b), −18 °C (c), and −20 °C (d) scale bars are 200 μm. (e–g) pore size distribution of PIC cryogels prepared at −16 °C (e), −18 °C (f), and −20 °C (g). (h) Average pore size of PIC cryogels prepared at −16/–18/–20 °C. Pore sizes we determined from z-stacks of CLSM images. Per condition, three cryogels were imaged, and for each cryogel, three *z*-stacks were analyzed to obtain an average pore size per cryogel. (i) Young's moduli of PIC cryogels prepared at −16/–18/–20 °C. Young's moduli were calculated from the stress strain curves that were obtained by compressing the cryogels. A derivative of the slope at 0.8 strain was taken to calculate Young’s moduli depicted in (i). (j) Swelling ratio of PIC cryogels prepared at −16/–18/–20 °C. (e–j) Data are represented as mean ± SEM, *N* = 3 or 4. (h–j) Statistical significance was tested with one-way ANOVA with posthoc Tukey’s multiple comparison test. Stars indicate significance levels * *P* ≤ 0.05, *** *P* ≤ 0.001, and ns is nonsignificant.

The cryogelation process is characterized by a balance between the cross-linking rate and the rate of ice crystal formation; to obtain a macroporous structure, the cross-linking rate should be slower than the rate of crystallization.^74−76^ Furthermore, too fast cross-linking can lead to formation of heterogeneous cryogel networks.^77^ Consequently, the temperature at which cryopolymerization takes place influences the nucleation and crystallization rate as well as the cross-linking rate, which means that the cryogelation temperature has a substantial impact on cryogel features such as pore size, structural homogeneity, and polymer wall thickness.^78^ Since slight variations in temperature can already significantly affect the cryogel,^68,79−81^ we prepared cryogels of similar composition at temperatures of **–16**, **–18,** and **–20 °C** (Figure 3a). We observed that cryogels prepared at **–16 °C** had a broader pore size distribution (Figure 3b–g) and a larger average pore size (32.8 μm) than cryogels prepared at **–18 °C** (18.2 μm) and **–20 °C** (17.0 μm) (Figure 3h). The thermal effects include contributions of the actual polymerization temperature but also cooling rates, since all samples start from 0 °C. Literature shows that a higher cooling rate leads to more ice nucleation and ice crystal formation, which gives rise to cryogels with smaller and more uniform pores.^78,82^ The difference in cryogel pore size and uniformity is obvious when cryogelation temperature decreased from **–16** to **–18 °C** but becomes less pronounced when comparing cryogels prepared at **–18** and **–20 °C**.

Mechanical characterization revealed that, at 80% strain (Figure S6), cryogels prepared at **–16 °C** showed the lowest stiffness (4.2 kPa) compared to those of the cryogels prepared at **–18 °C** (4.7 kPa) and **–20 °C** (6.9 kPa, Figure 3i). The pore size correlates with Young’s modulus; cryogels with smaller pores have a higher Young’s modulus. This correlation suggests that a smaller pore size results in a cryogel with higher compressive strength.^79,83−85^ At lower cryogelation temperatures, the volume of the nonfrozen microphase decreases due to the increased ice crystallization. As a result, the polymer concentration in the microphase increases, which in turn gives rise to cryogels with denser polymer walls.^54^

We also examined the effect of the cryogelation temperature on the swelling behavior of the prepared cryogels (Figure 3j). While all cryogels exhibited swelling behavior that is characteristic of highly interconnected macroporous gels, we observed a decrease in swelling ratio between cryogels prepared at **–16 °C** (3208%) and **–18 °C** (2215%). The cryogels that were prepared at **–18 °C** have smaller pores than the **–16 °C** cryogels and a higher Young’s modulus, which results in lower swelling.^78,81,86,87^ Interestingly, the swelling ratio increased for cryogels prepared at **–20 °C** (4137%) in comparison to the **–18 °C** cryogels, despite the fact that the **–20 °C** cryogels have slightly smaller pores and higher stiffness. We tentatively attribute this effect to the narrower pore size distribution for the **–20 °C** cryogel.^88^ Since the difference in pore size between the cryogels is statistically insignificant, it could be that the influence of pore size distribution on swelling behavior becomes distinct, explaining the observed increased swelling ratio for cryogels prepared at **–20 °C**.

Interestingly, the influence of the cryogelation temperature on the architectural and mechanical properties of PIC cryogels seems to reach an optimum at −20 °C. PIC cryogels that were prepared at **–22 °C** had a slightly smaller pore size than the cryogels prepared at **–20 °C** (Figure S7a–c) and displayed a significantly lower Young’s modulus (3.1 kPa, Figure S7d). The decrease in compressive strength can be explained by a compromised mechanical stability as a result of thinner polymer walls within the cryogel (Figure S7e).^79^ This finding is supported by analysis of the swelling ratio of the **–22 °C** cryogels, which is lower (2755%) than that for the **–20 °C** cryogels (Figure S7f). Both the **–20** and **–22 °C** cryogels have a similar polymer concentration, yet the pore walls of the **–22 °C** cryogels are thinner and thus have a higher polymer density, which negatively affects the swelling capacity.^55,86,89,90^ In summary, we stress that the cryogelation temperature is an easy parameter to manipulate key cryogel characteristics, such as architecture and mechanical properties, while keeping the cryogel composition unchanged.

### Polymer Concentration Influences the Mechanical Properties of PIC Cryogels

**Figure 4:**
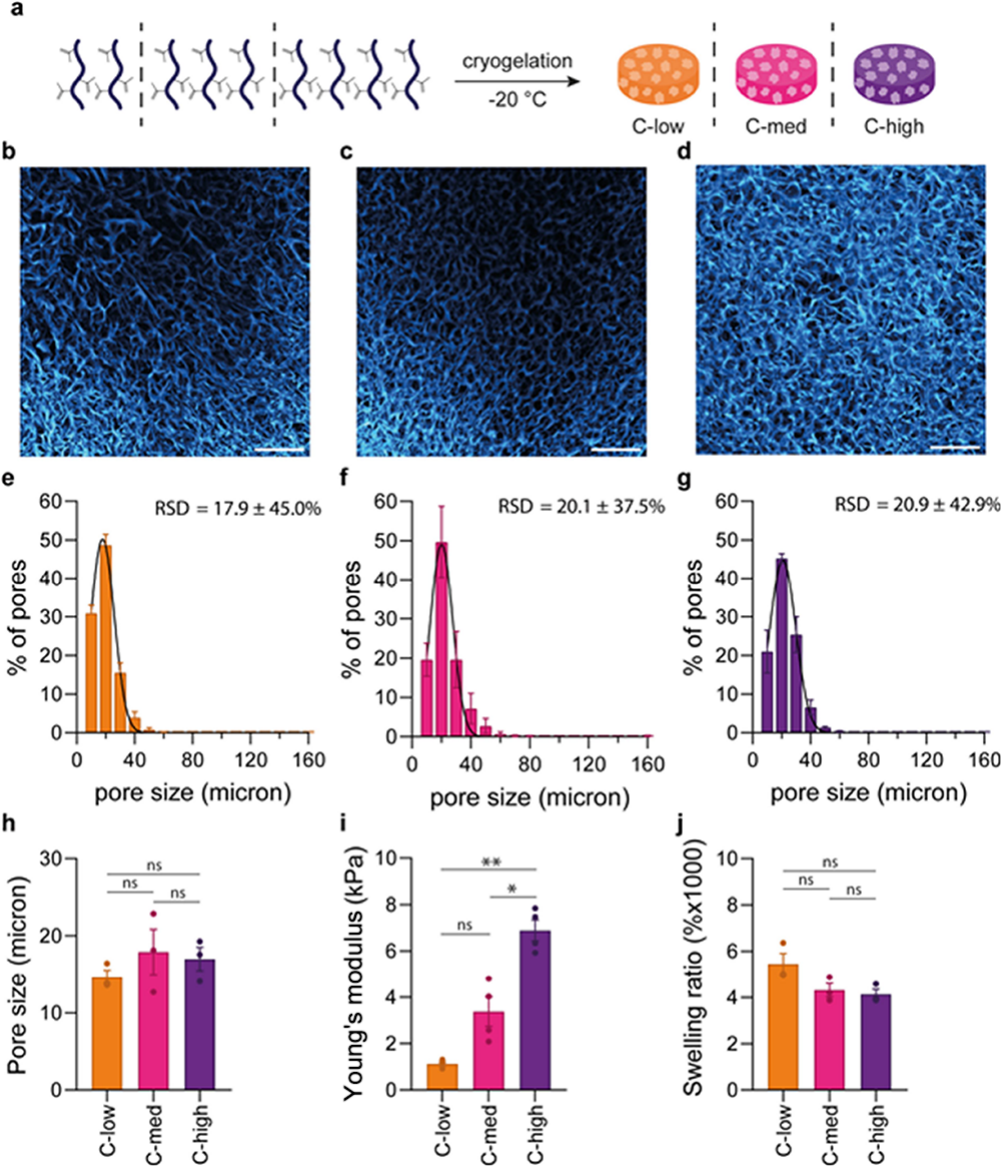
Polymer concentration influences mechanical properties of PIC cryogels. (a) Schematic overview of cryogel synthesis with different polymer concentrations. (b and c) confocal images showing the interconnected macroporous structure of PIC cryogels prepared with low (b), medium (c), and high (d) PIC concentration. Scale bars are 200 μm. (e–g) Pore size distribution of PIC cryogels prepared with low (e), medium (f), and high (g) PIC concentration. (h) Average pore size of PIC cryogels. Pore sizes we determined from z-stacks of CLSM images. Per condition, three cryogels were imaged, for each cryogel three z-stacks were analyzed to obtain an average pore size per cryogel. (i) Young's moduli of PIC cryogels prepared with low, medium and high PIC concentration. Young's moduli were calculated from the stress strain curves that were obtained by compressing the cryogels. A derivative of the slope at 0.8 strain was taken to calculate Young’s moduli depicted in (i). (j) Swelling ratio of PIC cryogels prepared with low, medium and high PIC concentration. (e–j) Data are represented as mean ± SEM, *N* = 3 or 4. (h–j) Statistical significance was tested with one-way ANOVA with posthoc Tukey’s multiple comparison test (h, j) or Welch ANOVA with posthoc Dunnet’s T3 multiple comparison test (i). Stars indicate significance levels * *P* ≤ 0.05, ** *P* ≤ 0.01, and ns is nonsignificant.

To determine how the properties of PIC cryogels can be tuned, we investigated the influence of the polymer concentration. In general, an increase in polymer concentration results in cryogels with smaller pores due to the decreased amount of free water available for ice crystallization.^69,86^ We prepared cryogels with low, medium, and high polymer concentrations (respectively, 3.75, 6.67, and 9.17 mg/mL), resulting in cryogels **C-low**, **C-med,** and **C-high**, respectively (Figure 4a). Here, we observed that polymer concentration had no significant influence on the pore size and pore size distribution. All cryogels have highly interconnected macroporous structures, narrow pore size distributions, and similar pore sizes: 14.6, 17.9, and 16.8 μm for **C-low**, **C-med,** and **C-high**, respectively. (Figure 4b–h). We hypothesize that the polymer concentration does not affect ice crystal formation rates, which yields similar cryogel architectures, albeit with higher densities of polymer walls for the higher concentration cryogels. It is worth noting that for most other cryogels, the polymer concentration is considerably higher than for PIC cryogels; while for PIC cryogels the highest polymer concentration is 1 wt %, polymer concentrations for other cryogels typically vary from 2 to 8 wt %.^79,91^

Next, we examined the influence of polymer concentration on their mechanical properties by subjecting cryogels **C-low**, **C-med,** and **C-high** to uniaxial compression tests (Figure 4i). **C-low** cryogels have Young’s modulus of 1.1 kPa, **C-med** cryogels of 3.4 kPa, and **C-high** cryogels of 6.9 kPa. There is a linear correlation between polymer concentration and Young’s modulus (Figure S8). The prepared cryogels displayed similar swelling behavior (Figure 4j). **C-low** cryogels had a slightly higher swelling ratio (5450%) than **C-med** (4320%) and **C-high** (4137%). A decreased polymer concentration affords cryogels with a lower density polymer walls, which results in a higher swelling ratio due to the increased flexibility of the structure.^55,86,89,90,92−94^ The absence of significant differences for swelling ratio and pore size between the cryogels further underlines the previously described argument that the swelling ratio of PIC cryogels mostly correlates to the porous structure of the cryogels. Based on these results, we note that we can employ polymer concentration to tune the mechanical properties of PIC cryogels without affecting pore size and porosity.

### Polymer Molecular Weight Influences the Mechanical Properties of PIC Cryogels

**Figure 5:**
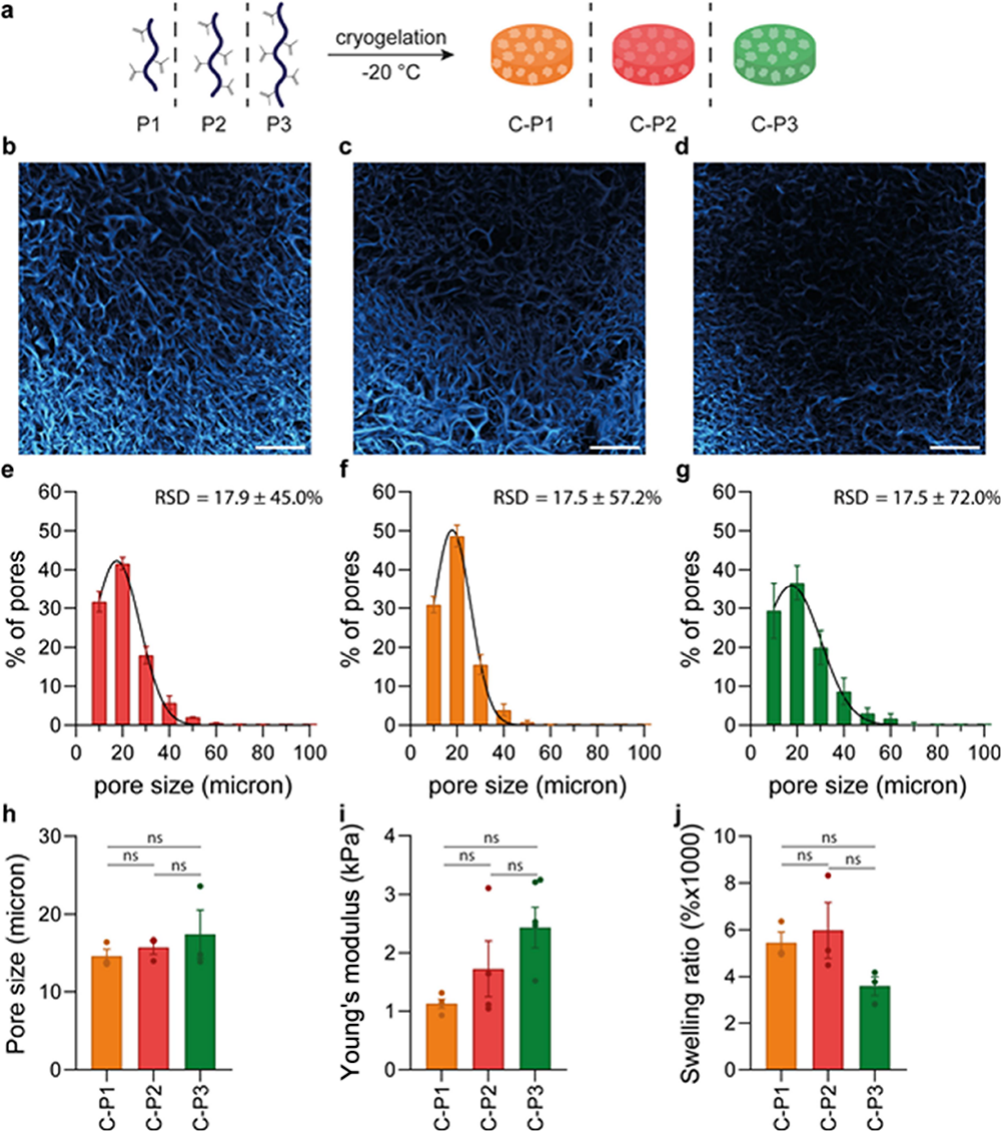
Polymer length influences mechanical properties of PIC cryogels. (a) Schematic overview of cryogel synthesis with different polymers. (b, c) Confocal images showing the interconnected macroporous structure of PIC cryogels prepared with P1 (b), P2 (c), and P3 (d). Scale bars are 200 μm. (e–g) Pore size distribution of PIC cryogels prepared with P1 (e), P2 (f), and P3 (g). (h) Average pore size of PIC cryogels. Pore sizes were determined from z-stacks of CLSM images. Per condition, three cryogels were imaged; for each cryogel, three z-stacks were analyzed to obtain an average pore size per cryogel. (i) Young's moduli of PIC cryogels prepared with P1, P2, or P3. Young's moduli were calculated from the stress strain curves that were obtained by compressing the cryogels. A derivative of the slope at 0.8 strain was taken to calculate Young’s moduli depicted in (i). (j) Swelling ratio of PIC cryogels prepared with P1, P2, or P3. (e–j) Data are represented as mean ± SEM, *N* = 3. (h–j) Statistical significance was tested with one-way ANOVA with posthoc Tukey’s multiple comparison test. ns is nonsignificant.

Besides the cryogelation temperature and polymer concentration, we find that the molecular weight of the polymers influences cryogel properties. Usually, at a fixed polymer concentrations, the use of polymers with lower molecular weights results in cryogels with larger pores.^55,56,95−97^ This phenomenon can be explained by the Mark–Kuhn–Houwink equation, which states that an increase in the molecular weight results in a decrease in the free water content in the polymer solution that is available for crystallization. As a result, cryogels with smaller pores and thicker polymer walls are generated.^56^ We used PIC scaffolds of increasing length (and molecular weight), **P1**, **P2,** and **P3**, at a constant concentration, to prepare cryogels **C–P1**, **C–P2,** and **C–P3**, respectively (Figure 5a).

We observed that the polymer molecular weight had no significant influence on pore size and pore size distribution. All cryogels have a highly interconnected macroporous structure and similar pore size distributions (Figure 5b–g). Additionally, all cryogels displayed a similar pore size of 14.6, 15.7, and 17.4 μm, for **C–P1, C-P2,** and **C–P3**, respectively. These results are in line with the previously reported trend for PIC hydrogels, where polymer molecular weight did not influence the hydrogel architecture.^19^ Our results, analogous to earlier reports of other materials^98,99^ contradict the common trend for cryogels. We hypothesize that, similar to that described previously, the ice crystal formation does not depend on the polymer molecular weight and thus gives cryogels with similar architectures in which polymer walls have higher density for the cryogels with higher molecular weight polymer scaffolds.

Young’s modulus of cryogels **C–P1**, **CP-2,** and **CP-3** was determined from the uniaxial compression tests, and we found that the molecular weight of the PIC scaffolds slightly influences the mechanical properties of the formed cryogels. **C–P1** cryogels shows Young’s modulus of 1.1 kPa, **C–P2** cryogels of 1.7 kPa, and **C–P3** cryogels of 2.4 kPa. The molecular weight between cross-links of a cryogel plays an important role in determining the mechanical strength. Cryogel networks with larger molecular weight between cross-links usually have a lower compressive strength than cryogels with smaller molecular weight between cross-links.^100^ We hypothesize that for **C–P3** the molecular weight between the cross-links is smaller than for **C–P1** and **C–P2**. Because of the larger polymer scaffold used, it could be that more cross-links are made in close proximity, resulting in a lower molecular weight between cross-links.

Additionally, the observed increase in compressive strength underlines the hypothesis that PIC scaffolds with high molecular weight are more tightly packed within the nonfrozen microphase than scaffolds with low molecular weight. All prepared cryogels displayed similar swelling behavior (Figure 5j). **C–P1** and **CP–2** cryogels had a swelling ratio slightly higher than that of **C–P3**, which can be explained by their somewhat lower mechanical strength. Cryogels with less stiff networks are capable of taking up more water than cryogels with a more compact, dense network.^55,56^ Together, we conclude that while the polymer molecular weight has some effect on cryogel features such as compressive strength and swelling ratio, the influence is moderate compared to the influence of cryogelation temperature or polymer concentration, which makes it a less effective parameter.

## Conclusions

In conclusion, we have successfully synthesized and utilized methacrylate-functionalized PIC scaffolds to prepare PIC cryogels. These cryogels have a highly interconnected macroporous structure and exhibit an excellent swelling behavior. We optimized the composition of PIC cryogels by fine-tuning the amount of cross-linking initiator and addition of a chain extender (HEMA), which resulted in cryogels with a more homogeneous porous architecture, substantial compressive strength, and high porosity. Cryogels with such features are highly desirable for biomedical applications, as their large pore size and high interconnectivity can facilitate cell migration, proliferation and metabolic activity.^101^

We investigated which variables in the cryogelation process can be used to tune the architectural and mechanical properties of PIC cryogels. The ability to tailor the properties of cryogels is of interest for biomedical applications as it allows us to optimize the material depending on their application. Cryogels for tissue engineering applications require different mechanical properties depending on which tissue is reconstituted. For instance, bone marrow cells require cryogel scaffolds with higher stiffness than skin cells.^102,103^ In line with the mechanical properties of specific human tissues, one may anticipate that PIC cryogels find application as artificial lymphoid or neural tissue.^104,105^ By systematically altering either cryopolymerization temperature, polymer concentration, or polymer molecular weight during the cryogelation process and analysis of the architectural and mechanical properties of the resulting cryogels, we found that we could not only alter the properties of PIC cryogels but also selectively tune their stiffness.

Decreasing cryopolymerization temperature from −16 to −20 °C reduced the pore size of PIC cryogels, due to the influence of temperature on the interplay between cross-linking and ice crystal formation. The decrease in the pore size consequently resulted in cryogels with a higher compressive strength. Interestingly, we observed a cutoff for this trend at a cryogelation temperature of −22 °C. Cryogels prepared at this temperature had only slightly smaller pores than cryogels prepared at −20 °C but displayed a significantly lower compressive strength. Altering the polymer concentration within the PIC cryogels affected their mechanical properties, but not their pore size and swelling capacities. Increasing the concentration of the PIC scaffold afforded cryogels with higher compressive strength while their architecture remained similar to cryogels with low PIC concentrations. This observation is rare, as in most cases an increase of polymer concentration results in cryogels with smaller pores due to the decreased amount of free water available for ice crystal formation.^55,56,69,86^ Finally, we found that altering the molecular weight of the PIC scaffolds only influenced the mechanical properties of the resulting cryogels slightly, while the pore size remained unaffected. Again, this observation contradicts what is commonly found in the literature, as for most cryogels pore size decreases when polymers with higher molecular weight are used, following the Mark–Kuhn–Houwink equation.^95−97^

Based on our findings, we conclude that PIC cryogels are tunable by simply changing the cryogelation temperature or the polymer concentration, which alters the architectural and mechanical properties. Uniquely, we can tune the compressive strength of PIC cryogels without affecting the pore size. Either by lowering the cryogelation temperature to −22 °C or by altering the polymer concentration. Such selective tuning of mechanical properties is highly useful for biomedical applications, as it well-known that the mechanical properties of the material surrounding cells influence cell behavior.^106,107^ Previous research with PIC hydrogels underlines that slight alterations in the mechanical properties can have a significant impact on cell proliferation and cell faith.^26,108^ Because PIC cryogels enable the decoupling of matrix stiffness from other variables such as pore size, they provide an interesting three-dimensional model system for biomedical research. The azide groups on the PIC scaffold facilitate post functionalization of the cryogel through click chemistry, which allows us to decorate the cryogel with a variety of biomolecules that are relevant for biomedical applications. As such, we conclude that PIC cryogels are useful new materials in the toolbox of biomedical scientists.

## Supporting Information Available

Synthesis scheme and experimental data (PIC and cryogel analysis) (PDF)
Cryogel confocal fluorescence microscopy z-stack (Video S1) (AVI)

## Supplementary Material

bm4c00086_si_001.pdf

bm4c00086_si_002.avi
