# Supplementary material for: Tailoring of Physical Properties in Macroporous Poly(isocyanopeptide) Cryogels
Source: Biomacromolecules. 2024 May 14;25(6):3464–74. doi: 10.1021/acs.biomac.4c00086 (PMC11170948; doi:10.1021/acs.biomac.4c00086)
Supplement: Supplementary file 1 — bm4c00086_si_001.pdf [file bm4c00086_si_001.pdf]

Electronic supplementary information for:

# Tailoring of physical properties in macroporous poly(isocyanopeptide) cryogels

Lotte Gerrits,[a,b] Bram Bakker,[a] Lynn D. Hendriks,[a] Sjoerd Engels,[a] Roel Hammink,*[c,d] Paul H. J. Kouwer*[a,b]

a. Institute for Molecules and Materials, Radboud University, Heyendaalseweg 135, 6525 AJ Nijmegen, The Netherlands. Institute for Chemical Immunology, Nijmegen 6525 GA, Netherlands.

b. Institute for Chemical Immunology, Nijmegen 6525 GA, Netherlands.

c. Department of Medical BioSciences, Radboudumc, Geert Grooteplein 26, 6525 GA Nijmegen, The Netherlands.

d. Division of Immunotherapy, Oncode Institute, Radboud University Medical Center, Nijmegen 6525 GA, Netherlands.

*Email: Roel.Hammink@radboudumc.nl; Paul.Kouwer@ru.nl

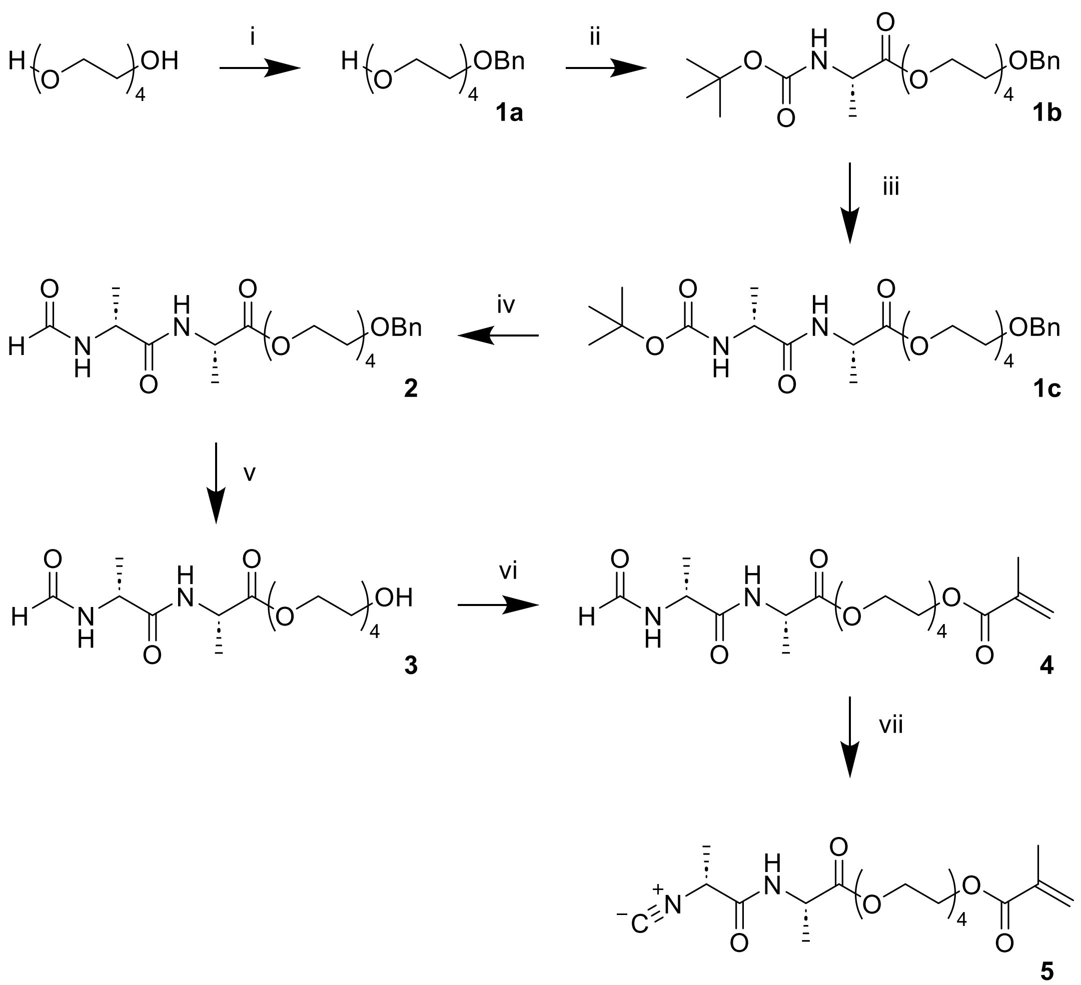


**Scheme 1. Synthesis route towards methacrylate functionalized isocyanide monomer 5.** Key: (i) NaH, BnBr, THF, r.t., 72 h (ii) Boc-L-Ala, DMAP, DCC, DCM, 0 °C → r.t., 19 h (iii) 1. HCl (4M in dioxane), DCM, r.t.. 3 h 2. Boc-L-Ala, DMAP, DCC, HOBt, DCM, 0 °C → r.t., 19 h (iv) 1. HCl (4M in dioxane), DCM, r.t.. 2 h 2. HCOONa, Ethyl formate, reflux, 19 h. (v) Pd/C, $H_2$, EtOH, r.t., 18 h (vi) Sodium methacrlylate, EDC.HCl, DIPEA, DMAP, DCM, 0 °C → r.t., 18 h (vii) Burgess reagent, DCM, r.t., 6 h.

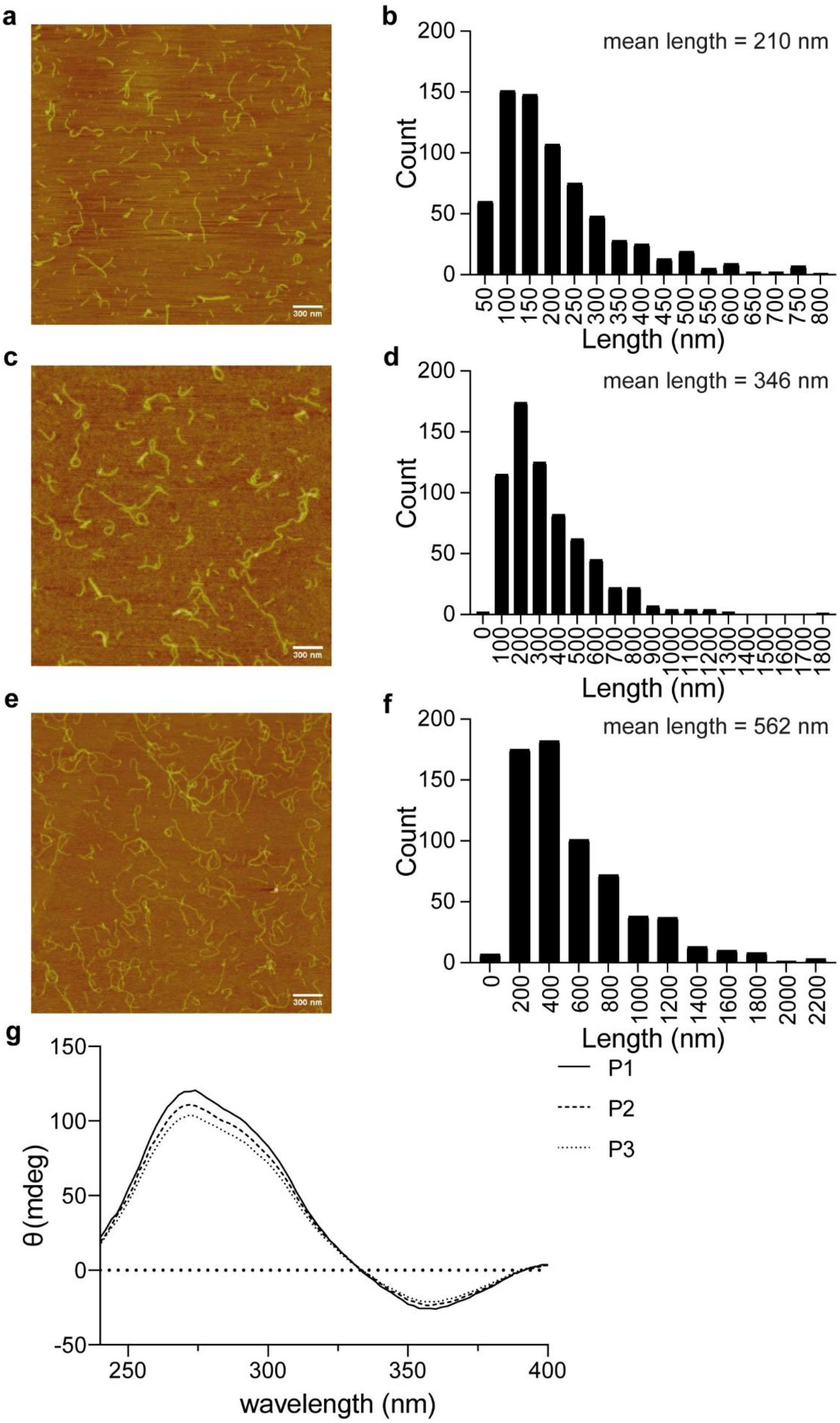


**Figure S1. Characterization of PIC-methacrylate P1, P2 and P3.** (a) Representative AFM image of P1 polymers on a mica surface. Scale bar 300 nm. (b) Histogram depicting the length distribution of P1. Average polymer length is 210 ± 138 nm. (c) Representative AFM image of P2 polymers on a mica surface. Scale bar 300 nm. (d) Histogram depicting the length distribution of P2. Average polymer length is 346 ± 231 nm. (e) Representative AFM image of P3 polymers on a mica surface. Scale bar 300 nm. (f) Histogram depicting the length distribution of P3. Average polymer length is 562 ± 390 nm. (i) Circular Dichroism spectroscopy of P1, P2 and P3 (0.2 mg/ml in PBS)

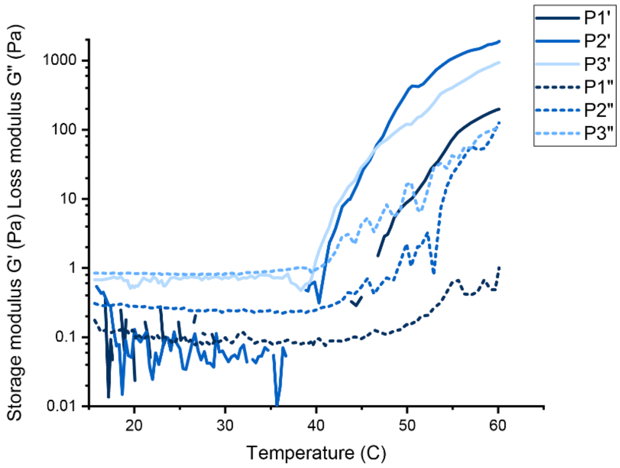


**Figure S2** Temperature sweeps of **P1**, **P2** and **P3** (4 mg/ml in MQ). Solid lines represent storage moduli *G'*, dashed lines represent loss moduli *G"*.

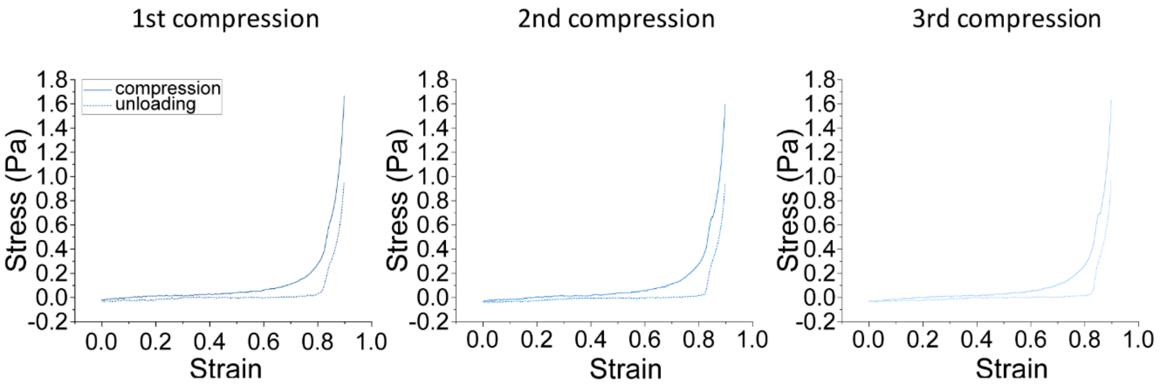


**Figure S3.** Stress strain curves from three consecutive rounds of uniaxial compression and unloading of PIC cryogels.

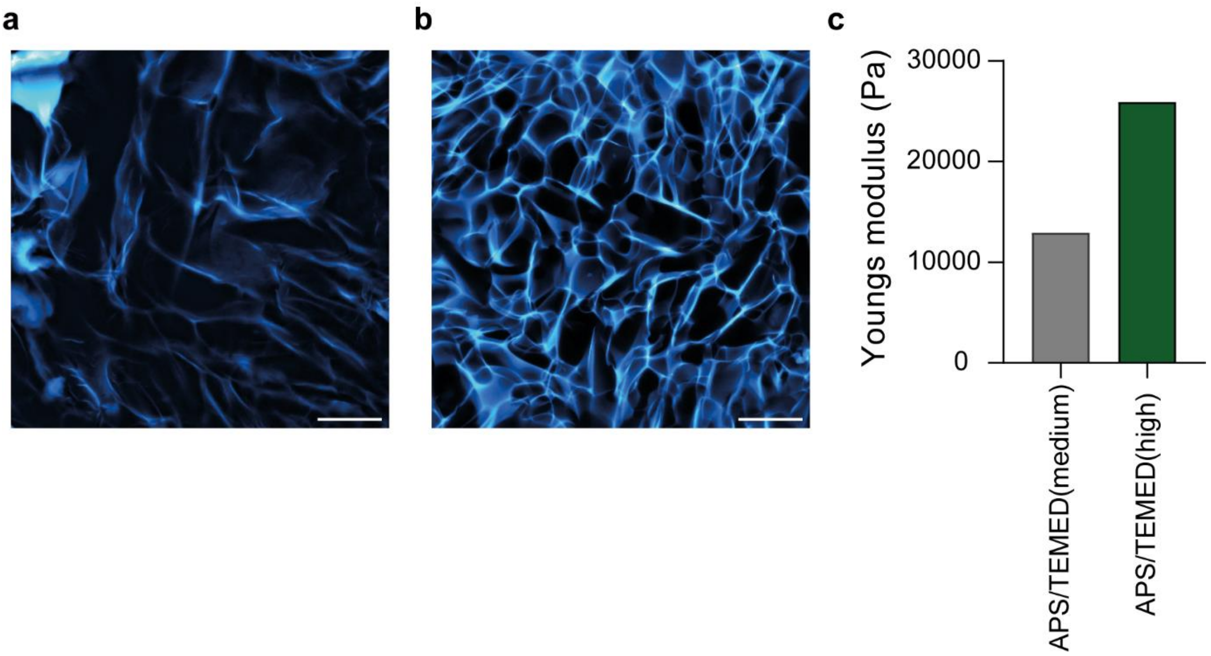


**Figure S4.** Varying the amount of APS/TEMED influences architectural and mechanical properties of PIC cryogels. (a-b) confocal images showing the interconnected macroporous structure of PIC cryogels prepared with medium (a) and high (b) amounts of APS/TEMED. Scale bars are 200 µm. (c) Youngs moduli of PIC cryogels prepared with medium and high amounts of APS/TEMED. Youngs moduli were calculated from the stress strain curves that were obtained by compressing the cryogels. A derivative of the slope at 0.80 strain was taken to calculate Young's moduli depicted in (c). (a-c) For cryogels APS/TEMED(medium), respective concentrations of 0.015 mM APS and 0.007 mM TEMED were used, while for cryogels APS/TEMED(high), concentrations of 0.02 mM APS and 0.01 mM TEMED were used.

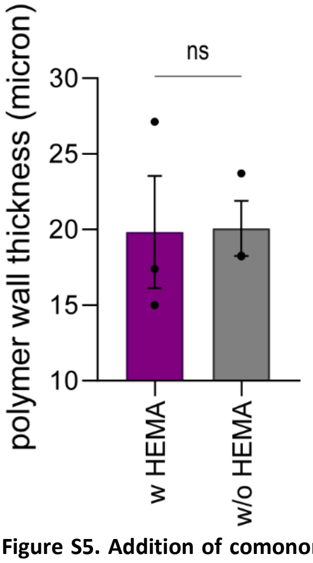


**Figure S5. Addition of comonomer (HEMA) influences architectural and mechanical properties of PIC cryogels.** Average polymer wall thickness of PIC cryogels. Wall thicknesses were determined from z-stacks of CLSM images. Per condition, 3 cryogels were imaged, for each cryogel 3 z-stacks were analyzed to obtain an average polymer wall thickness per cryogel. All data is represented as mean + standard error of the mean. Statistical significance was tested with an unpaired t-test, ns, non significant.

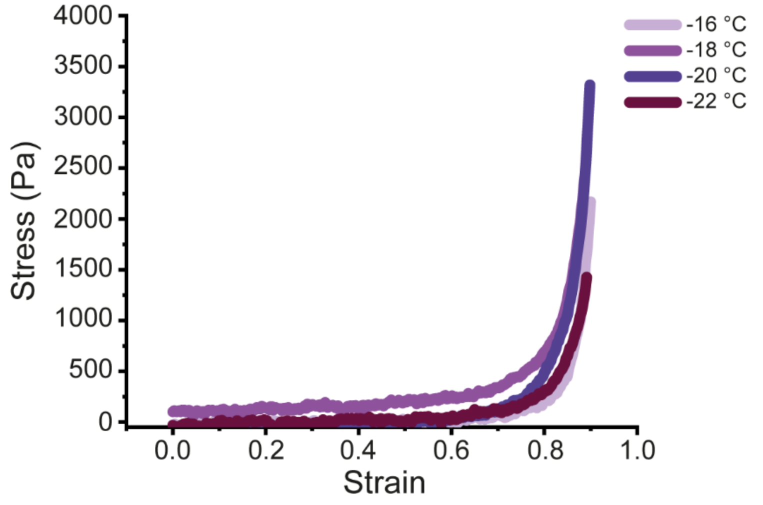


**Figure S6.** Stress-strain curves of PIC cryogels prepared at **–16**/**–18**/**–20**/**–22** °C. Curves were obtained by uniaxial deformation of the cryogels on a rheometer.

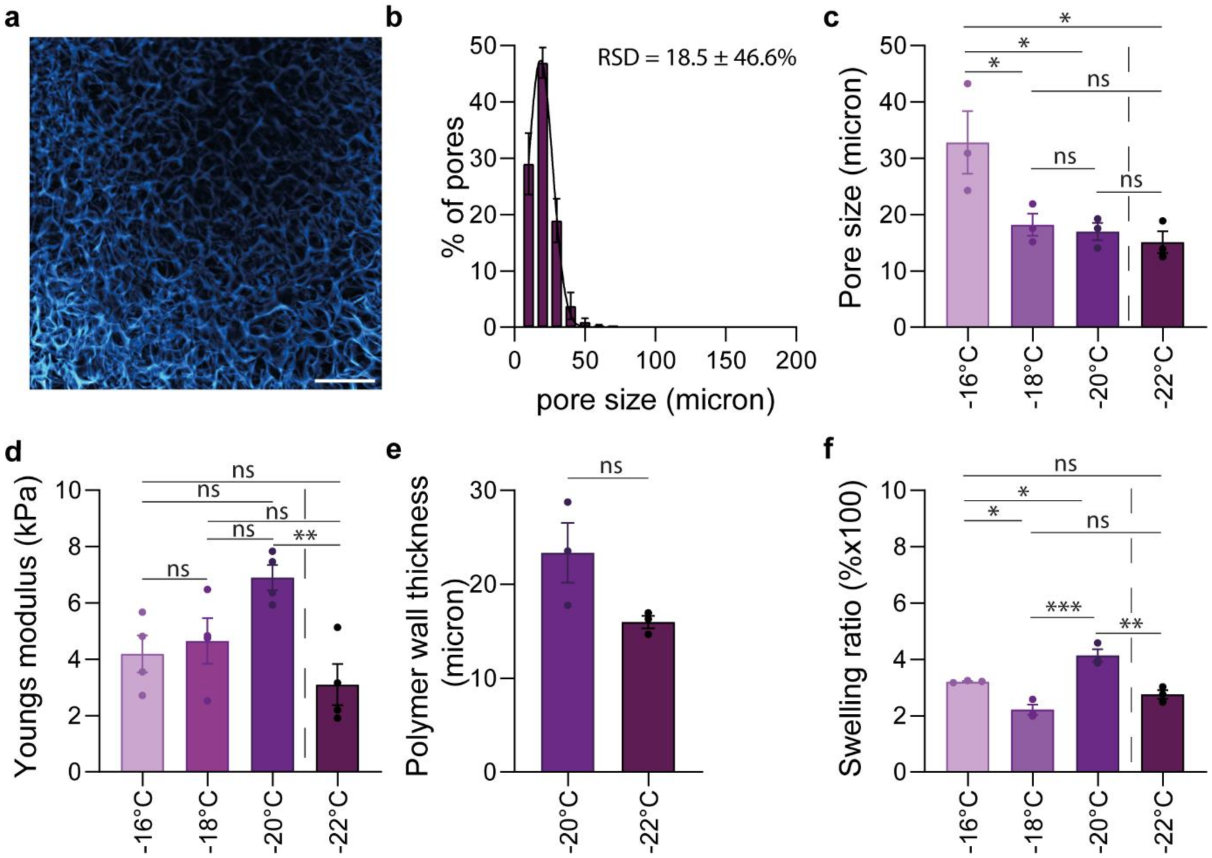


**Figure S7. Cryogelation temperature influences architectural and mechanical properties of PIC cryogels.** (a) Confocal image showing the interconnected macroporous structure of a PIC cryogels prepared at –22 °C. Scale bars are 200 µm. (b) Pore size distribution of PIC cryogels prepared at –22 °C. (c) Average pore size of PIC cryogels prepared at **–16**/**–18**/**–20**/**–22** °C. Pore sizes we determined from z-stacks of CLSM images. Per condition, 3 cryogels were imaged, for each cryogel 3 z-stacks were analyzed to obtain an average pore size per cryogel. (d) Youngs moduli of PIC cryogels prepared at **–16**/**–18**/**–20**/**–22** °C. Youngs moduli were calculated from the stress strain curves that were obtained by compressing the cryogels. A derivative of the slope at 0.8 strain was taken to calculate Young's moduli depicted in (d). (e) Average polymer wall thickness of PIC cryogels prepared at **–20** and **–22** °C. Polymer wall thickness was determined from z-stacks of CLSM images. Per condition, 3 cryogels were imaged, for each cryogel 3 z-stacks were analyzed to obtain an average polymer wall thickness per cryogel. (f) Swelling ratio of PIC cryogels prepared at **–16**/**–18**/**–20**/**–22** °C. All data is represented as mean + standard error of the mean.

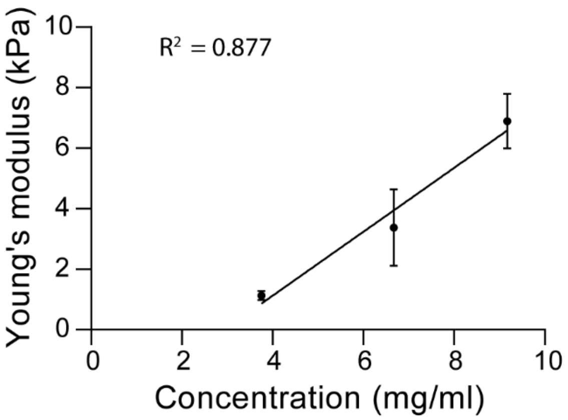


**Figure S8. Linear correlation between polymer concentration and Young's moduli of the compressed cryogels at 80% strain.** Young's moduli were calculated from the stress strain curves that were obtained by uniaxial deformation of the cryogels. Every data point is depicted as mean ± SD, where n=4.
